# Bacterial Protein Toll-Like-Receptor Agonists: A Novel Perspective on Vaccine Adjuvants

**DOI:** 10.3389/fimmu.2019.01144

**Published:** 2019-05-29

**Authors:** Sudeep Kumar, Raju Sunagar, Edmund Gosselin

**Affiliations:** ^1^Department of Immunology and Microbial Diseases, Albany Medical College, Albany, NY, United States; ^2^Ella Foundation, Genome Valley, Hyderabad, India

**Keywords:** adjuvant, TLR agonist, TLR, antigen presenting cells, cell-mediated immunity, vaccine

## Abstract

Adjuvants have been used in vaccines for over a century, however, the search for safe and effective vaccine adjuvants continues. In recent decades toll-like-receptor (TLR) agonists have been investigated as potential vaccine adjuvants. In this regard, the majority of the currently investigated TLR agonists are non-protein microbial components such as lipopolysaccharides, oligonucleotides, and lipopeptides. On the other hand, a growing number of studies reveal that TLR signaling and immune responses can be activated by numerous bacterial proteins. However, their potential roles as adjuvants have been somewhat overlooked. Herein, we discuss several such bacterial proteins which exhibit adjuvant properties, including the activation of TLR signaling, antigen presenting cell maturation, pro-inflammatory cytokine production and adaptive immune response. The protein nature of these TLR agonists presents several unique features not shared by non-protein TLR agonists. These properties include the amenability for modifying the structure and function as necessary for optimal immunogenicity and minimal toxicity. Protein adjuvants can be genetically fused to protein antigens which ensure the co-delivery of adjuvant-antigen not only into the same cell but also in the same endocytic cargo, leading to more effective activation of innate and adaptive immune response.

## Introduction

### Vaccine and Adjuvants

Since its discovery more than a century ago vaccines continue to save millions of lives and prevent many more from the debilitating effects of numerous infectious diseases each year (1). Following Edward Jenner's successful use of a Cowpox virus to protect humans from Smallpox (2, 3), several live attenuated vaccines have been developed such as measles, mumps, rubella, rotavirus, influenza, tuberculosis, cholera, and typhoid (4). Live attenuated vaccines are comprised of weakened forms of pathogenic microbes which cause limited infections, but nevertheless induce long-lasting protection (3). On the other hand, many killed vaccines have also been developed, which completely lose the ability to cause infections (4). However, both live-attenuated and killed-vaccines pose significant safety concerns due to the potential reversion to pathogenic forms or inadequate inactivation. (3). The next generation of vaccines, known as subunit-vaccines have further improved the safety profile of vaccines due to the use of acellular microbial components including toxoids, polysaccharides and proteins (3, 4). However, the enhanced safety profile of antigens used in subunit-vaccines is associated with poor immunogenicity, a deficiency which adjuvants are required to overcome (5). In addition to enhancing the immunogenicity, adjuvants also reduce the total amount of antigens and the number of immunizations required to achieve an adequate level of protective immunity (6).

### Mechanism of Action of Adjuvants

**Figure 1:**
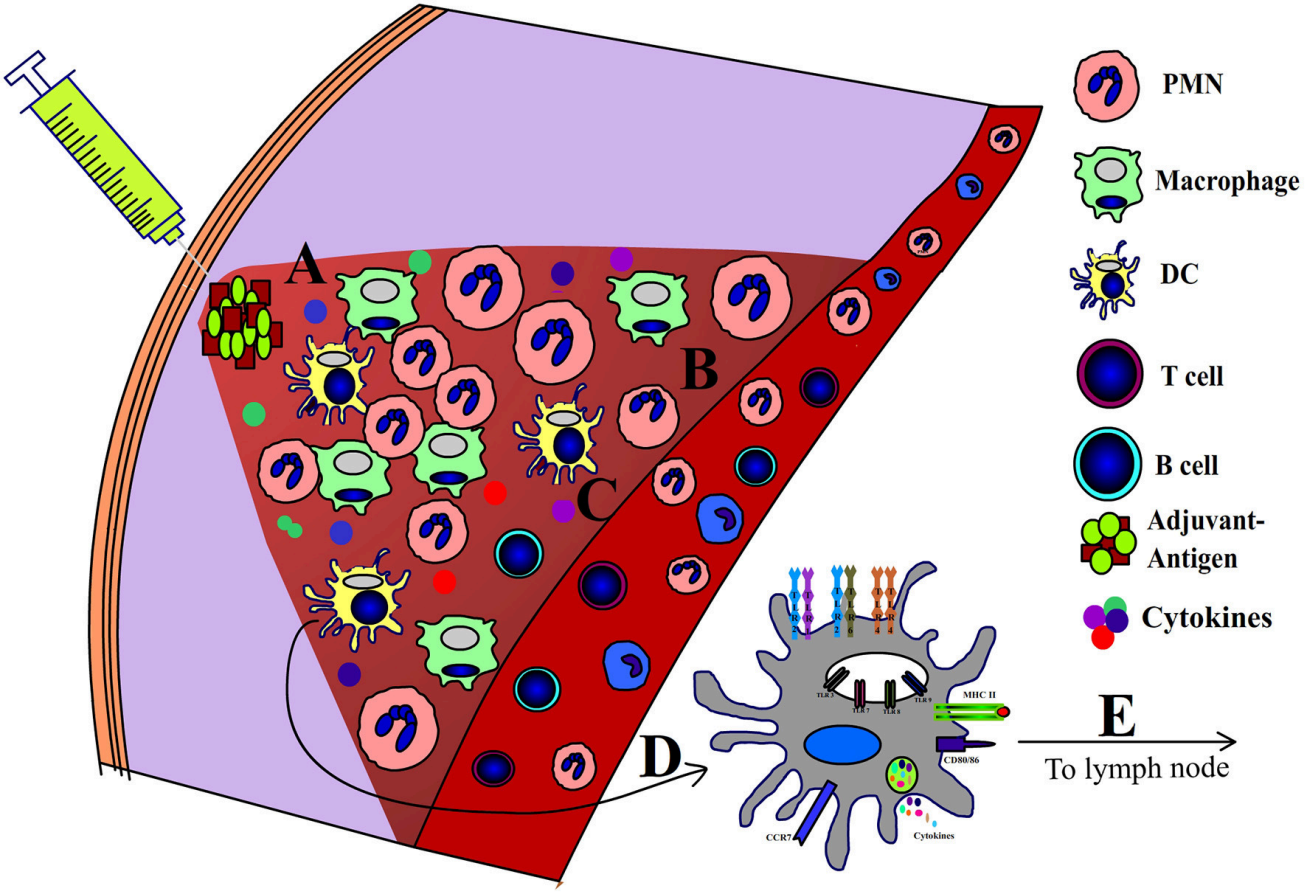
Mechanism of action of adjuvants. Following administration adjuvants induce a local **(A)** pro-inflammatory niche which is characterized by **(B)** influx of leukocytes, **(C)** pro-inflammatory cytokines, **(D)** activation of APCs, and **(E)** migration of APCs to draining lymph nodes.

Adjuvants are defined as molecules or formulations that enhance the efficacy of vaccines without directly participating in the protective immunity. Although the mechanism of action of adjuvants is not fully understood, most adjuvants exhibit many shared immunological features. First and foremost, adjuvants induce a local pro-inflammatory environment at the site of administration. Inflammation is mediated by several pro-inflammatory cytokines and chemokines including: IL-1β, IL-6, TNF-α, and IL-12 (7). Adjuvants also induce recruitment of various innate immune cells including neutrophils, macrophages and DCs (7–10). Adjuvants activate professional antigen presenting cells (APCs) and promote the uptake of antigens (10–12). When exposed to various adjuvants, APCs increase the expression of MHC class II (MHCII), and co-stimulatory molecules (CD40, CD80/CD86) (9, 10, 13). The MHCII- peptide complex provides the first signal required for CD4^+^ T-cell activation, while the second necessary signal is relayed by the engagement of CD28 to CD80/CD86 (10) (Figure 1). Notably, the activated APCs possess all the molecular mediators to facilitate both (First and Second) signals required to activate naïve T cells. Importantly, adjuvants also induce cytokine production by APCs, which influence the T cell's polarization toward Th1, Th2, or Th17 phenotypes. Specifically, IL-12 promotes the Th1 phenotype (14), IL-4 and IL-10 can promote the Th2 phenotype (14), and the combined effects of TGF-β and IL-6 promote the Th17 phenotype (15, 16). Also, it is now well recognized that cytokines secreted by activated APCs are required to overcome peripheral-tolerance controlled by CD4^+^CD25^+^ Treg cells, which limits the adaptive immune responses to antigens (17). Adjuvants also enhance the expression of CCR7 on APCs, which promote their migration to the draining lymph nodes, wherein processed antigens are presented to the naïve T cells (9).

### Current Challenges in Vaccine Adjuvants

Since the discovery of the immune enhancing properties alum several new adjuvants have been developed including MF59, AS03, AS01, and AS04 (18, 19) and these adjuvants have been being used in more than 100 vaccine formulations including influenza, polio, hepatitis A, *B pertussis*, and tetanus vaccines (20). However, several challenges related to adjuvants still remain that require continued efforts (21) to develop novel adjuvants. For example, the lack of an effective adjuvant is hindering the prospects of an effective vaccine against various forms of cancer. Moreover, mucosal vaccines are considered superior to the parenteral vaccines in combating mucosal infection, however, the lack of effective mucosal adjuvants limit the development of mucosal subunit-vaccines (21, 22). Similarly, vaccines for elderly and immuno-compromised populations pose other challenges; although the elderly are less responsive to vaccination, the immune-compromised can be more susceptible to the live attenuated vaccines (23, 24).

### Bacterial Protein TLR Agonists as Adjuvants

Due to their role in self/nonself-differentiation (25) and their ability to induce APC maturation, TLR agonists are considered promising adjuvant candidates (26). In fact, a number of TLR agonists including Pam3CSK4, Pam2CSK4, MPLA (LPS derivative), CpG, PolyI:C, and flagellin are currently being tested/used as adjuvants (20, 27, 28).

In recent decades there have been numerous studies showcasing the immunomodulatory properties of microbial proteins that parallel the activities of adjuvants. However, except for the bacterial flagellins and porins, their potential role as adjuvants has not been well-investigated or realized. In this article we discuss the immunological attributes of various bacterial protein TLR agonists (BPTAs), specifically related to adjuvant properties induced via TLR signaling. While searching for potential protein adjuvants we focused our attention on bacterial proteins that exhibit one or more of the following properties: (1) engage and activate TLR2 or TLR4 signaling; (2) induce pro-inflammatory cytokines; (3) up-regulate the expression of co-stimulatory molecules on APCs; (4) induce antibody mediated; or (5) induce cell-mediated immunity. We have limited our search to the TLR2, TLR4, and TLR5 agonists, because their respective receptors are expressed on the surface of APCs. Due to the cell surface expression these receptors can also be utilized to target the antigens to APCs by fusing the antigens to various TLR2/4/5 agonists.

Bacterial flagellins also fall in the category of BPTAs, however, they are well-recognized TLR5 dependent vaccine adjuvants, and thus we are not discussing it further in this article. Several recent articles (29–31) provide comprehensive review of the role of flagellins in TLR5 dependent adjuvant activity.

### TLR2 Dependent BPTAs

TLR2 forms heterodimers with TLR1 and TLR6, which recognize tri-acylated lipoproteins and di-acylated lipoproteins respectively. Apart from lipoproteins TLR2 also respond to a diverse array of microbial patterns including peptidoglycan, lipoteicoic acid, lipoarabinomannan, zymosan, and phospholipomannan (32), suggesting a promiscuous nature of TLR2. Emerging evidences suggest that TLR2 signaling is also activated by a variety of bacterial proteins (Table 1).

**Table 1:** A list of BPTAs, which exhibit adjuvant potential.

	**Bacteria**	**TLR agonist**	**Innate activity**	**Adaptive immune response**	**References**
**TLR2 AGONISTS**					
1	*Brucella abortus*	BCSP31	Pro-inflammatory cytokines	Th1	(33)
2	*Bordetella pertussis*	FHA	Langerhans cell recruitment	Serum and mucosal IgG and IgA	(34, 35)
3	*Chlamydia trachomatis*	MOMP	IL-6 and IL-8	ND	(36)
4	*Fusobacterium nucleatum*	FomA	B cell activation, IL-6	Th2 type	(37)
5	*Mycobacterium tuberculosis*	MymA (Rv3083), ESAT6	Macrophage activation, pro-inflammatory cytokines (IL-6, TGF-β, IFN-β)	Th1, Th17	(38–40)
6	*Neisseria meninigitidis*	PorB	APC activation, Ag presentation,	CD8^+^ T cell	(41, 42)
7	*Staphylococcus aureus*	PVL	Macrophage pro-inflammatory cytokines	ND	(43)
8	*Shigella dysenteriae*	Porin	B cell activation, Pro-inflammatory cytokines	IgM,IgG	(44–46)
9	*Shigella flexneri*	OmpA, 34 kDa MOMP	B cell activation, pro-inflammatory cytokines, Macrophage activation,	IgG, IgA, Th1 mediated immunity	(47–50)
10	*Streptococcus pneumoniae*	PepO	Macrophage, pro-inflammatory cytokines	ND	(51)
11	*Vibrio cholerae*	OmpU	M1 polarization, pro-inflammatory cytokines	ND	(46)
**TLR4 AGONISTS**					
12	*Brucella abortus*	Lumazine synthase, Omp16, Omp19, BCSP31	DC maturation, DC recruitment to lymph nodes, pro-inflammatory cytokines,	Th1, Th17, and Mucosal immunity	(33, 52–54)
13	*Mycobacterium paratuberculosis*	CobT, RpfE, Rv0652, HBHA	DC maturation, and cytokine secretion	Th1, Th17, CD4^+^ and CD8^+^	(55–58)
14	*Neisseria meninigitidis*	NhhA	Macrophages, Pro-inflammatory cytokines	Th1 and Th17	(59)
15	*Streptococcus pneumoniae*	DnaJ, Pneumolysin, ΔA146 Pneumolysin	DC maturation, Macrophage activation, IL-12 secretion	IgG, IgA & IL-17A Th1, Th17	(60–64)

The outer membrane proteins (OMPs) of *Shigella flexneri* (Outer membrane protein A and major outer membrane protein) (47–49) and *Chlamydia trachomatis* (major outer membrane protein) (36) are known to induce TLR2 signaling. These OMPs elicit pro-inflammatory cytokines IL-12p70, TNF-α, and IL-6 (36, 49, 50), induce maturation markers on the APCs (MHCII, CD80, CD86) (48–50), and orchestrate humoral (IgG and IgA) (50) and cell mediated (Th1 polarized) immune responses (48). Bacterial pore forming proteins (porins) are another class of outer membrane proteins implicated in innate immunity and the activation of TLR2 signaling. The porins from *Shigella dysenteriae* (44, 45, 65), *Vibrio cholerae* (OmpU) (46), *Neisseria lactamica* (PorB) (66), *Neisseria meninigitidis* (41, 42), and *Fusobacterium nucleatum* (FomA) (37) have been identified as inducers of TLR2 signaling. These porins induce pro-inflammatory cytokines (37, 45), activate APCs (37, 45), and induce Th1 type (44) and humoral immune responses (37, 65). Panton-Valentine leukocidin (PVL) is a pore forming peptide from *Staphylococcus aureus* that directly binds to TLR2 and modulates the expression of 29 genes in murine alveolar macrophages, and induces innate immune responses and pro-inflammatory cytokines (43).

Some other bacterial proteins also exhibit TLR2 agonist function. For example, the early-secreted-antigen (ESAT6) of *M. tuberculosis* induces secretion of IL-6 and TGF-β by dendritic cells in a TLR2-dependent manner (39). Moreover, Chatterjee et al. showed that ESAT6 induces Th17 response, which plays an important role in protection against *M. tuberculosis* infection (39). The recombinant Brucella-cell-surface-protein-31 (rBCSP31) from *Brucella abortus*, which can interact with both TLR2 and TLR4, induces TNF-α, IL-6, and IL12-p40. Li et al. further demonstrated that TLR2 and TLR4 deficient macrophages secrete lower levels of cytokines compared to the wild type macrophages when treated with the rBCSP31. The rBCSP31 also induces Th1 type immune response in a TLR2 and TLR4-dependent manner, and protects against *B. abortus* infection (33). The mycobacterial protein MymA is a TLR2 agonist, which induces APC function of the human monocyte derived macrophages, including up-regulation of CD40, CD80, CD86, and HLA-DR expression, and secretion of TNF-α and IL-12. Moreover, MymA also polarizes the host immune response toward Th1 by increasing the secretion of IFN-γ (38). *S. pneumoniae* proteins DnaJ and pneumolysin (Ply) are known to activate TLR4 signaling but not TLR2 signaling. However, DnaJ-ΔA146Ply, which is a genetic fusion of DnaJ and a ply mutant (ΔA146Ply), induces protection of mice in a TLR2-dependent manner. Furthermore, TLR2 deficiency reduces the ability of DnaJ-ΔA146Ply to induce Th1 type immune response (60). Another protein from *S. pneumoniae*, endopeptidase O (PepO) exhibits TLR2 and TLR4 agonist properties. Specifically, the recombinant-PepO results in a significant increase of cytokines production and neutrophils infiltration in the lungs of wild type mice compared to that of TLR2 or TLR4 knockout mice. The recombinant-PepO also induces TNF-α, IL-6, CXCL-1, and CXCL-10 in peritoneal exudate macrophages (PEMs) in a TLR2 and TLR4-dependent manner (51).

### TLR4 Dependent BPTAs

LPS is the best-characterized TLR4 ligand. Upon LPS engagement the TLR4 signaling pathway results in the activation of pro-inflammatory response and maturation of APCs. In addition, TLR4 signaling by LPS also activates T-cell mediated immune response. MPLA which is a less toxic version of lipid A is being utilized as adjuvant in several vaccine formulations due to its ability to effectively activate TLR4 signaling (67). Interestingly, emerging evidences suggest that TLR4-signaling can also be activated by various BPTAs, which also play important roles in the generation of protective immune responses (Table 1).

Several pneumococcal proteins show potent TLR4 activation capacity including Ply, PepO, and DnaJ. Ply, PepO (51) and DnaJ induce TNF-α, IL-6, CXCL-1, and CXCL-10. Ply also confers TLR4 dependent protection against *S. pneumoniae* infection (61). Recombinant DnaJ (rDnaJ) induces maturation of DCs by activating the TLR4 pathway. The rDnaJ treated DCs polarize naïve CD4^+^ T cells to Th1 and Th17 in a TLR4 dependent manner (62). Fusion of DnaJ and a Ply mutant (ΔA146Ply-DnaJ and DnaJ-ΔA146Ply) induce B and T cell dependent protection against *S. pneumoniae* infection, while DnaJ-ΔA146Ply induces IL-4, IFN-γ and IL-17A in a TLR4 dependent manner (63).

Similarly, *Brucella* spp. also harbor various TLR4 dependent BPTAs including Lumazine synthase (BLS), outer membrane protein-16 (Omp16) and outer membrane protein-19 (Omp19). BLS is capable of forming stable oligomers and stimulating DCs to increase the expression of CD40, CD80, CD86, and MHCII in a TLR4 dependent manner. BLS also increases the expression of several cytokines and chemokines and triggers the recruitment of DCs *in vivo*, depending on TLR4 signaling (52). Interestingly Omp16 and Omp19 are lipoproteins and it is conceivable that the associated lipid moieties induce TLR2 pathway, however, the un-lipidated recombinant Omp16 (rOmp16) and recombinant Omp19 (rOmp19) both exhibit potent immunogenicity in a TLR4 dependent manner. The rOmp16 and rOmp19 both induce DC maturation by up-regulating the expression of CD40, CD80 and CD86 *in vitro* as well as *in vivo*. Moreover, the rOmp16 and rOmp19 both exhibit mucosal immunogenicity as their oral delivery induces protective immunity against *B. abortus* (53, 54). Furthermore, the rOmp-19 also induces Th1 and Th17 type adaptive immune response in mice (54).

*M. tuberculosis* derived resuscitation-promoting-factor-E (RpfE), heparin binding hemagglutinin (HBHA), and the 50S ribosomal protein (Rv0652) exhibit TLR4 dependent BPTA activity. RpfE, HBHA, and Rv0652 induce DC maturation by increasing the surface expression of maturation markers CD40, CD80/CD86, and MHC class I/II and the production of IL-6, IL-1β, IL-23p19, IL-12p70, and TNF-α in a TLR4 dependent manner (55–57). HBHA also promote DC migration by increasing the expression of CCR-7. RpfE facilitates CD4^+^ T cell differentiation to Th1 and Th17 through modulation of dendritic cell function. The HBHA treated and antigen pulsed DCs induce antigen-specific tumor cell cytotoxicity in a murine thymoma model and prolong the survival of vaccinated mice (56). The Rv0652 pulsed DCs activate and polarize naïve CD4^+^ and CD8^+^ T cells to secrete IFN-γ, and induce T cell-mediated cytotoxicity. Moreover, Lee et al. also demonstrated that immunization with Rv0652-stimulated and ovalbumin (OVA)-pulsed DCs induces a potent OVA-specific CD8^+^ T cell response, restrict tumor growth, and promote long-term survival (57).

*Mycobacterium paratuberculosis* derived protein CobT activates the TLR4 pathway and induces DC maturation. The CobT-stimulated DCs also polarize naïve CD4^+^ and CD8^+^ T cells to secrete IFN-γ and IL-2, but not IL-4 and IL-10. Furthermore, the CobT-stimulated DCs induce T cell proliferation (58).

## Molecular Mechanism of TLR-BPTA Interaction

TLRs belong to the leucine-rich-repeat (LRR) family, which interact with a variety of ligands including nucleic acids, peptidoglycan, lipo-peptides and lipids. Interaction with their corresponding ligands results in TLR dimerization, which triggers recruitment of adaptor proteins to the cytosolic Toll IL-1 receptor (TIR) domain, thereby relaying downstream signaling. Models based on the crystal structures of TLR2/1 and TLR2/6 bound to their corresponding ligands suggest that the ligands participate in forming the necessary bridge for dimerization. Two of the three lipid chains of the triacyl-lipopeptides are embedded in the TLR2 while the third one is inserted into a hydrophobic pocket of TLR1, thus facilitating dimerization of TLR2/TLR1 (68–70). The ligand binding occurs at the convex region of both the TLRs (68–70). Similarly, diacyl-lipopeptides signals via TLR2/6 heterodimer, wherein the lack of interaction between the third lipid chain and TLR6 appear to be compensated by higher hydrophobic interaction between TLR2 and 6 (68, 69, 71). TLR4 on the other hand does not directly bind to its ligand LPS, rather its interaction with LPS is mediated by another protein MD-2. MD-2 binds to TLR4 primarily via hydrophilic interface at the concave surface of TLR4 forming MD2-TLR2 heterodimer. Five of the six lipid chains of the *E. coli* LPS are completely buried inside the MD-2-TLR4 while the sixth one participates in bridging the dimerization with the other TLR4 (68, 69). This pattern of dimerization based signaling also occurs in TLR3, suggesting that the dimerization is an essential step in TLR signaling which triggers the cytosolic TIR domains to recruit adaptor molecules, such as MyD88, MAL, TRIF, and TRAM, which then facilitate downstream signaling (68, 69).

To date, there is no model or crystal structure showing the mechanism of TLR2 and TLR4 signaling by proteinaceous ligands. TLR5 does interact with bacterial flagellin proteins, however there is no data on its mechanism of interaction. MD-2 binding to TLR4 does suggest a possible mechanism of TLR4 interaction with proteinaceous ligands. Moreover, upon looking at the LRR family proteins, it is evident that the proteinaceous ligands for this family of receptors is quite common (72). The concave surfaces provide largely hydrophilic interaction, suitable for interaction with proteinaceous ligands (72). Thus, it is conceivable that many BPTAs interact in a similar fashion and trigger dimerization of their respective TLRs and downstream signaling. Moreover, BPTAs interacting with the same TLR may share structural similarities, which enable their interaction with the same TLR. However, more studies will be required to understand the molecular mechanism behind the BPTAs- TLR interaction as well as the ensuing downstream signaling.

## Perspective

### Physical Linkage Between Antigens and TLR Agonists and the Immunogenicity of Antigens

The generation of immunity against pathogens and tolerance toward self-antigens relies on the remarkable ability of the immune system to distinguish self from non-self antigens. Pathogens first encounter the components of the innate immune system, which are endowed with germline encoded pattern recognition receptors that recognize conserved molecular patterns in microbes. Moreover, APCs employ multiple mechanisms that control any aberrant immune response against self-antigens while allowing the generation of immune response to foreign antigens (73), and the TLRs play key role in this process.

**Figure 2:**
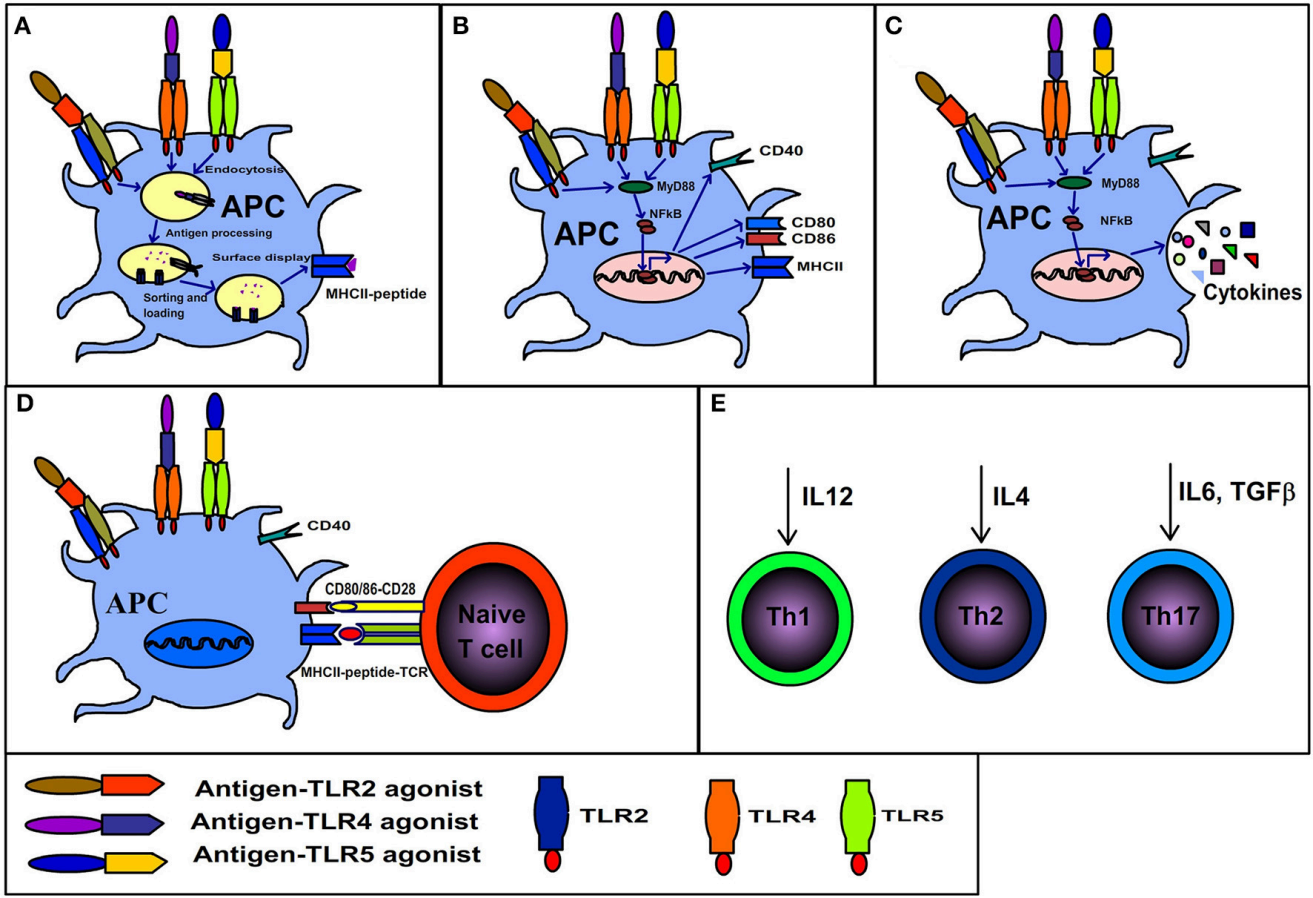
Immunological consequences of interaction between antigen-TLR agonist fusion protein and antigen presenting cells. **(A)** Antigens physically linked to TLR agonists are endocytosed along with the related receptor, the TLR dependent signaling in endosomes results in rapid maturation of phagosome, antigen processing, MHCII invariant chain processing, loading of processed antigens to MHCII and display of MHCII-peptide. **(B)** TLR signaling initiated by binding of antigen-TLR agonist results in maturation of APCs and surface expression of co-stimulatory molecules (CD80/86). **(C)** TLR signaling elicits cytokine secretion. **(D)** The APCs displaying co-stimulatory markers and MHCII-peptide activate naïve T cells. **(E)** The synergistic effects of A, B C and D cumulate in the generation of polarized T cells (Th1, Th2, or Th17) depending on the kind of cytokine secreted by APCs in response to respective TLR ligands.

At the cell surface of APCs, the TLR engagement enhances endocytosis of TLR bound ligands. While, in the endosomal compartment TLR signaling affects phagosomal maturation by enhanced acidification and fusion of the MHCII compartment. The phagosomal maturation allows the controlled proteolytic activity, thereby generating peptides from antigens associated with the TLR compartment (74–76). Additionally, the TLR dependent proteolysis of MHC-II associated invariant chain enables MHCII to accept peptides (Figure 2). On the other hand, the non-TLR bearing endosomes such as those originating from the phagocytosis of apoptotic cells are directed to terminal degradation. Therefore, the autologous antigens originating from dying-cells when phagocytosed by APCs are not loaded and presented by MHCII (77–79). Moreover, the TLR signaling induces the expression of co-stimulatory molecules necessary for the activation of naïve T cells. Consequently, the antigens that share the TLR compartment are more efficiently presented to CD4^+^ T cells (77, 78, 80, 81). Overall, it shows that the TLR based recognition is a key mechanism that regulates the generation of immune response against the microbial antigens and maintenance of tolerance/ignorance toward self-molecules. It is also evident from the above discussion that antigen-TLR agonist linkage potentiates CD4^+^ T cell response. Importantly, due to their protein nature BPTAs can be efficiently fused to protein antigens by genetic engineering. While antigen- adjuvant linkage cannot be achieved with many non-protein adjuvants and even require cumbersome procedures for some.

### Overcoming the Challenges of Vaccine Adjuvants With BPTAs

BPTAs can effectively solve many problems related to the current approaches of adjuvants. As discussed above, BPTAs that activate TLR2 and TLR4 exhibit several core properties of vaccine adjuvants. BPTAs induce pro-inflammatory cytokines *in vitro* and *in vivo* and induce co-stimulatory markers on macrophages and DCs. *In vivo* BPTAs induce recruitment of macrophages, DCs and neutrophils, which is a hallmark of adjuvant function. The APCs stimulated by BPTAs activate naïve T cells and polarize toward Th1 (33, 53, 57) Th2 (37), or Th17 (40, 54). BPTAs also elicit cell-mediated immune response to co-administered antigens, thereby solving the problem of poor cell-mediated immunity induced by currently adopted adjuvant approaches (41, 42, 55). Studies have shown that many BPTAs, specifically the TLR4 based agonists, can be used as immuno-therapeutic vaccines against cancer (40, 57). BPTAs efficiently induce mucosal immune responses including secretory IgG and IgA (34, 54). Many BPTAs can potentially enhance immune responses of vaccines in the elderly population, although it has not been tested yet. Importantly, the BPTAs can be efficiently modified by genetic engineering to enhance safety for administration in immuno-compromised individuals. Proteins are biocompatible, thus BPTAs mitigate the problems of biocompatibility related to non-protein based adjuvants. Interestingly, in the case of polysaccharide conjugate vaccines, protein-based adjuvants can be used as both carrier protein as well as adjuvant. Similarly, the protein structure can be easily manipulated to generate desired characteristics including the higher immunogenicity to cognate antigens and minimal toxicity. This level of control over structure and function cannot be attained with non-protein adjuvants. Moreover, antigen-adjuvant fusion proteins can be produced using recombinant expression systems; consequently a separate production and formulation of antigens and adjuvants will no longer be required which will reduce the time and cost of vaccine manufacturing.

## Author Contributions

SK and RS collected data related to this article. SK, RS, and EG wrote the paper. SK and EG edited the paper.

### Conflict of Interest Statement

The authors declare that the research was conducted in the absence of any commercial or financial relationships that could be construed as a potential conflict of interest.
